# High resolution MRI-based radiomic nomogram in predicting perineural invasion in rectal cancer

**DOI:** 10.1186/s40644-021-00408-4

**Published:** 2021-05-26

**Authors:** Yan-song Yang, Yong-juan Qiu, Gui-hua Zheng, Hai-peng Gong, Ya-qiong Ge, Yi-fei Zhang, Feng Feng, Yue-tao Wang

**Affiliations:** 1 Department of Radiology, Affiliated Tumor Hospital of Nantong University, Nantong, 226001 Jiangsu Province China; 2 Department of Nuclear Medicine, The Third Affiliated Hospital of Soochow University, No.185, Juqian Street, Changzhou, 213003 Jiangsu Province China; 3 Department of Pathology, Affiliated Tumor Hospital of Nantong University, Nantong, 226001 Jiangsu Province China; 4 GE Healthcare, Shanghai, 210000 China

**Keywords:** Rectal cancer, Magnetic resonance imaging, Radiomics, Nomogram, Perineural invasion

## Abstract

**Background:**

To establish and validate a high-resolution magnetic resonance imaging (HRMRI)-based radiomic nomogram for prediction of preoperative perineural invasion (PNI) of rectal cancer (RC).

**Methods:**

Our retrospective study included 140 subjects with RC (99 in the training cohort and 41 in the validation cohort) who underwent a preoperative HRMRI scan between December 2016 and December 2019. All subjects underwent radical surgery, and then PNI status was evaluated by a qualified pathologist. A total of 396 radiomic features were extracted from oblique axial T2 weighted images, and optimal features were selected to construct a radiomic signature. A combined nomogram was established by incorporating the radiomic signature, HRMRI findings, and clinical risk factors selected by using multivariable logistic regression.

**Results:**

The predictive nomogram of PNI included a radiomic signature, and MRI-reported tumor stage (mT-stage). Clinical risk factors failed to increase the predictive value. Favorable discrimination was achieved between PNI-positive and PNI-negative groups using the radiomic nomogram. The area under the curve (AUC) was 0.81 (95% confidence interval [CI], 0.71–0.91) in the training cohort and 0.75 (95% CI, 0.58–0.92) in the validation cohort. Moreover, our result highlighted that the radiomic nomogram was clinically beneficial, as evidenced by a decision curve analysis.

**Conclusions:**

HRMRI-based radiomic nomogram could be helpful in the prediction of preoperative PNI in RC patients.

## Introduction

Colorectal cancer (CRC) is a frequently diagnosed tumor in clinical practice globally. It is a leading cause of cancer-related mortality, and about 40% of these tumors are rectal cancer (RC) [1, 2]. In the USA, an increasing number of RC cases are being diagnosed in < 50-year-old, and this proportion has increased from 13.9 to 15.2% from 2004 to 2015, and younger patients usually present with more advanced disease [3].

Perineural invasion (PNI) is a pathologic feature wherein cancer cells invade the neural structures, and the primary malignant tumor spreads along the nerve sheaths. PNI is an under-recognized route of metastatic spread [4, 5]. A growing body of evidence, including systematic review and meta-analysis, has shown that the presence of PNI is correlated with significantly poor prognosis [5–11].

PNI is, therefore, an indubitable prognostic factor in CRC cases. According to the national comprehensive cancer network (NCCN) guideline for RC, more detailed information of PNI should be reported in the pathologic evaluation of RC [12]. PNI status should also be evaluated [III, A] according to the European society for medical oncology (ESMO) clinical practice guidelines for RC. Moreover, perineural invasion/extensive extramural vascular invasion close to the mesorectal fascia can sufficiently reflect the necessity of postoperative chemoradiotherapy, if such a therapy is not given preoperatively [13]. Similarly, PNI status is a Class I recommendation of pathological diagnosis for RC patients after radical surgery, and postoperative chemotherapy is recommended for stage II patients (Level 1A evidence) according to the Chinese guidelines for RC [14].

Both Zhou Y et al. [8] and Liebig et al. [10] confirmed that the prognosis of stage II PNI-positive patients was equal to or worse than that of stage III patients. Therefore, just like lymph node metastasis, the status of PNI should also be considered a high-risk factor in selecting adjuvant treatment plans. Thus, it was necessary to assess the status of PNI through preoperative imaging.

As a newly emerging approach that can decode tumor phenotype on non-invasive medical imaging, increasing attention has been paid to radiomics recently. The usual steps are described as follows: (a) region-of-interest (ROI) of target lesions is delineated on medical images, and then high-throughput quantitative features are extracted, (b) the correlation between these quantitative features and tumor heterogeneity is analyzed, and a predictive model is established; and (c) the predictive model is validated to support clinical decision making [15–17].

MRI of RC, especially high-resolution magnetic resonance imaging (HRMRI), can be used to precisely assess many critical findings, such as circumferential resection margin (CRM) status, existence of extramural vascular invasion (EMVI), depth of extramural invasion, existence of discontinuous extramural vascular spread/deposits, and existence of mucin. These high-risk characteristics not restricted to T and N stages, may support clinical decision making and stratify RC patients into distinct prognostic groups. Therefore, MRI has a critical function in the pre- and post-treatment evaluation of RC [18, 19].

In our current work, we established and validated a radiomic nomogram that incorporated the radiomic signature, HRMRI findings, and clinical risk factors for individualized preoperative prediction of PNI in RC patients.

## Materials and methods

### Patients

Ethical approval was obtained for this retrospective analysis, and the need for informed patient consent was waived.

**Fig. 1:**
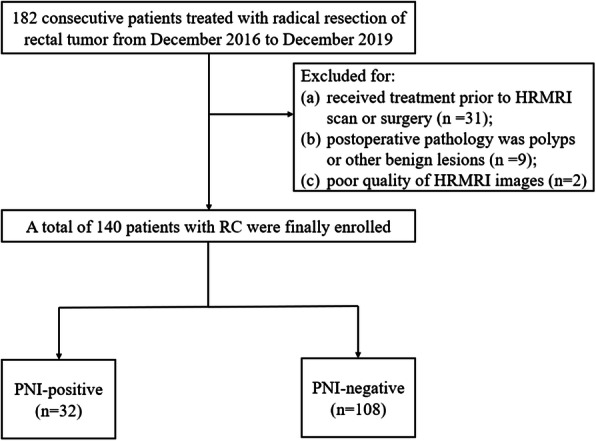
Flow chart of patient selection. RC = rectal cancer; PNI = perineural invasion

In this study, 182 consecutive subjects treated with radical resection of rectal tumor from December 2016 to December 2019, were included in the analysis. The inclusion criteria of our study were set as follows: (a) HRMRI was performed within 1 week before surgery, and the result indicated suspicious RC and (b) serum levels of carbohydrate antigen 19–9 (CA19–9) and carcinoembryonic antigen (CEA) were tested at our hospital within 1 week prior to surgery. The exclusion criteria were set as follows (Fig. 1): (a) patients who received treatment (radiotherapy, chemotherapy, or chemoradiotherapy) prior to HRMRI scan or surgery (*n* = 31), (b) the results of postoperative pathology showed polyps or other benign lesions rather than RC (*n* = 9), and (c) poor quality of HRMRI images which could not be used to accurately delineate lesions (*n* = 2). Finally, 140 RC patients were selected for our study and randomly assigned to the training and validation cohorts with a ratio of 7:3.

Information on baseline clinical risk factors, including age, gender, CA19–9, and CEA, were extracted from medical records. Levels of CA19–9 and CEA were examined via routine blood tests within 1 week prior to the operation.

### HRMRI protocol [18, 20]

All enrolled patients were scanned within 1 week before surgery by a 3.0-T MR (Magnetom Verio, Siemens Healthcare, Erlangen, Germany) with an 8-channel phased-array coil. The coil center was placed at the level of the pubic symphysis. Two-dimensional (2D) fast spin-echo (FSE) T2-weighted sequence without fat suppression was used with the following parameters: slice thickness, 3 mm; interval, 0.3 mm; TR/TE, 2500–3500 ms/100 ms; FOV, 18 cm × 18 cm; pixel matrix, 320× 320; and echo train length, 29. Sagittal localizing T2-weighted FSE images were acquired, and the oblique axial and coronal T2WI were achieved perpendicular and parallel to the long axis of the RC, respectively. Then an axial T1-weighted fast gradient echo sequence and contrast-enhanced T1 weighted scanning was conducted. The total HRMRI scanning time was about 20 min.

### HRMRI and pathological evaluation

The HRMRI findings, including primary site, longest diameter, MRI-reported tumor stage (mT-stage), MRI-reported lymph node status (mLN-status), CRM, and EMVI of the tumor, were retrospectively evaluated by two experienced radiologists (Reader 1 and Reader 2, with 14 and 9 years of experience in abdomen MRI, respectively) independently without knowing the pathological results, and any conflict of opinion was resolved by another senior radiologist with 24 years of experience. The primary site was classified as low, middle, and high according to the distance between the primary site and the anal verge. The mT-stage was divided into T1-T2 and T3.

In this study, malignant lymph node was recognized on HRMRI using the following criteria [18, 19]: (a) the short-axis diameter of suspicious lymph nodes measured on HRMRI ≥9 mm; (b) short-axis diameter between 5 and 8 mm, with more than two morphologically suspicious features (including irregular border, round shape, and heterogeneous signal); (c) short-axis diameter < 5 mm, with irregular border, round shape, and heterogeneous signal; and (d) all mucinous lymph nodes which showed T2WI high signal (any size). These standards are widely accepted and applied in routine clinical practice.

As histologic grade can be achieved via pre-surgery biopsy, it was counted as one clinical risk factor in our study and divided into well-, moderately-, and poorly differentiated tumor [21].

The PNI status of postoperative pathology was retrospectively re-evaluated by one pathologist with 20 years of experience in pathology blinded to the radiological findings.

### Segmentation of ROI and extraction of radiomic features

**Fig. 2:**
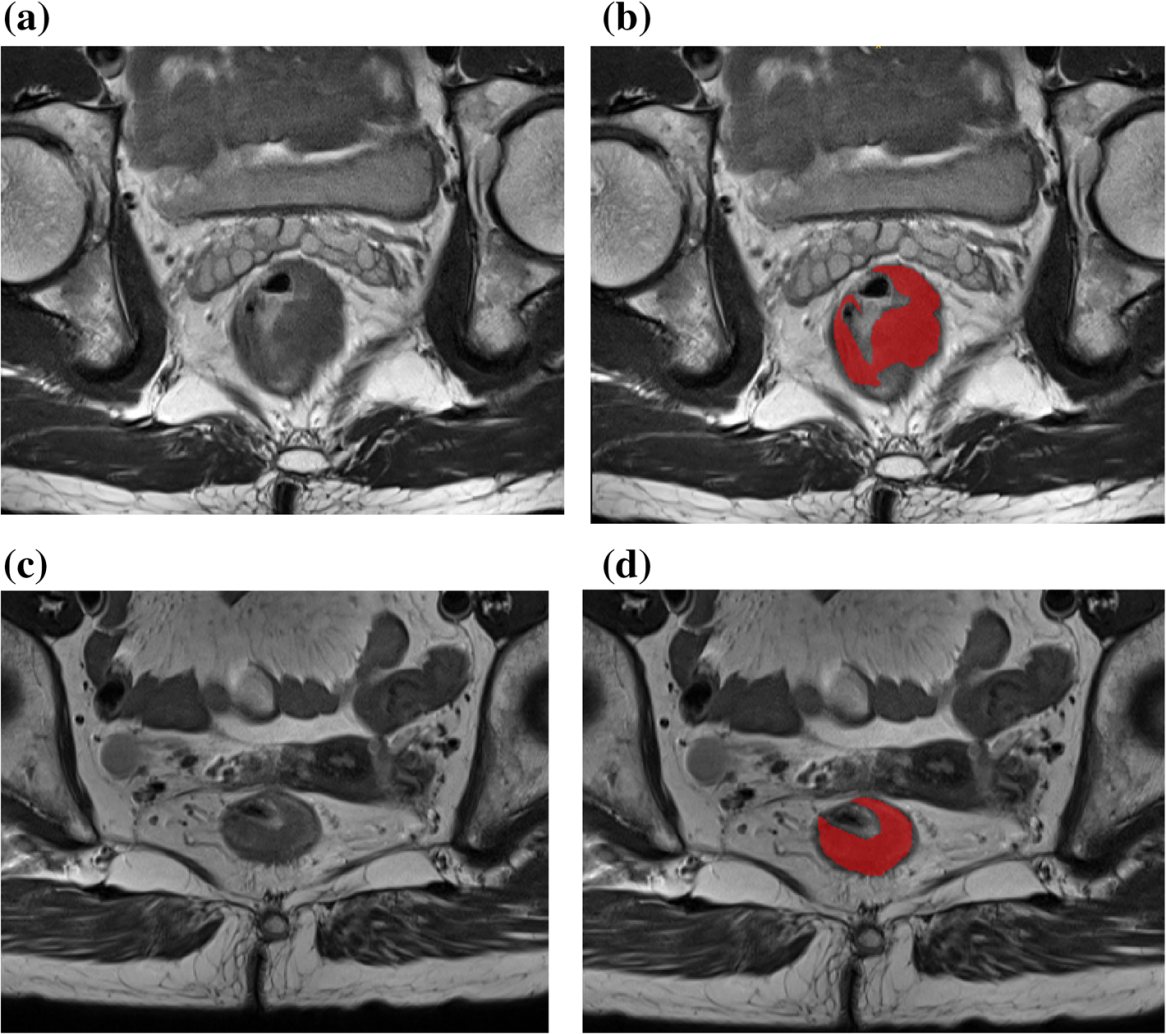
Tumor segmentation on RC HRMRI images by ITK software. **a**-**b** A 62-year-old male patient with RC, red regions indicate the ROI, and postoperative pathology confirmed PNI; **c**-**d** A 69-year-old female patient with RC, red regions indicate ROI and postoperative pathology confirmed PNI negative

Tumor ROI was semi-automatically sectioned in the largest cross-sectional area on oblique axial T2-weighted HRMRI (Fig. 2) by the ITK-SNAP software (www.itksnap.org). The reproducibility of the intra-observer and inter-observer segmentation was validated by Reader 1 and Reader 2. Radiomic features, including histogram, form factor, haralick, gray-level size zone matrix (GLSZM), gray-level co-occurrence matrix (GLCM), and run-length matrix (RLM), were determined by AK software (Artificial Intelligence Kit V3.0.0.R, GE Healthcare). A total of 396 imaging features, including 42 histogram features, 9 form-factor features, 10 haralick features, 11 GLSZM features, 144 GLCM features, and 180 RLM features, were isolated from each case before feature selection, and the detailed information of these features are included in the Supplementary Data. Isolated texture features were standardized to remove the data unit limits of each feature.

### Feature selection and construction of radiomic signature

The dimensionality reduction was conducted as follows: radiomic features were initially screened by the maximum correlation and minimum redundancy (mRMR) method, then, the least absolute shrinkage and selection operator (LASSO) was conducted to select an optimized subcohort of features to construct the final model, and lastly, logistic regression was applied to the selected features to build the radiomic signature, and the radiomic score (Rad-score) for each patient was calculated. The area under the curve (AUC) was employed to test the predictive accuracy of the Rad-score in both the training and validation cohorts.

### Construction and assessment of the radiomic nomogram

In the training cohort, Wilcoxon signed-rank test was applied to compare the difference of the continuous clinical features and HRMRI findings between PNI and non-PNI groups, such as age, CA19–9, CEA, longest diameter; chi-square test was applied to the categorical variables such as gender, histologic grade, primary site, mT-stage, mLN-status, CRM, and EMVI, and the factors with *p* < 0.1 were analyzed in a univariate logistic regression to choose the independent clinical or HRMRI finding predictors. Subsequently, multivariable logistic regression analysis was applied to the independent clinical risk factors, HRMRI findings, and Rad-score, followed by the establishment of the predictive nomogram. A calibration curve was employed to calibrate the nomogram. Furthermore, receiver operating characteristic (ROC) curves were employed to assess the discriminatory ability of the nomogram.

### The intra-observer and inter-observer agreements

An intra-class correlation coefficient (ICC) was employed to assess the intra-observer and inter-observer agreements of feature extraction. A total of 30 random patients were initially chosen for ROI segmentation and feature isolation. The segmentation of ROI was conducted independently by two senior radiologists. Intra-observer ICC was determined by comparing two extractions of Reader 1. Inter-observer ICC was determined by comparing the extraction of Reader 2 and the first extraction of Reader 1. An ICC greater than 0.75 indicated good agreement, and the segmentation of remaining images was carried out by Reader 1 [22, 23].

### Statistical analysis

R software (version 3.5.1; http://www.Rproject.org) was employed to perform the statistical analyses. Synthetic Minority Oversampling Technique (SMOTE) was used to amplify the small sample data sets to get a balanced data distribution of the with or without PNI groups in the training cohort to construct the model [24]. The ‘mRMRe’ package was used to perform mRMR to initially screen the radiomic features. The ‘glmnet’ package was used to select the best feature cohort and perform the LASSO regression analysis to construct a radiomic signature. ROC analysis was conducted based on the ‘pROC’ package to evaluate the usefulness of the model in PNI prediction. The ‘ModelGood’ package was used for calibration analysis of the model. The ‘rmda’ package was used to plot decision curves and validate the clinical value of the model. Other statistical analyses were carried out using SPSS 22.0. The level of statistical significance was set at *P* < 0.05.

## Results

### Demographics and clinical findings

A total of 140 RC cases, including 90 males and 50 females (mean age, 64 years; range, 34–86 years), were included in this retrospective analysis based on the above-mentioned selection criteria. Patients were randomly assigned into the training (*n* = 99) and validation (*n* = 41) cohorts after classifying the patients in PNI and non-PNI groups. Postoperative pathology confirmed that 32 patients had PNI (22.9%), and 108 patients had no PNI. Table 1 shows that there was a significant difference in variables (mT-stage, longest diameter, and EMVI) between patients with PNI and those without PNI in the training cohort, while no significant difference was found in other variables.

**Table 1:** Characteristics of patients in the training and testing cohorts

Characteristics	Training cohort (*n* = 99)			Testing cohort (*n* = 41)		
	With PNI (*n* = 23)	Without PNI (*n* = 76)	*P* value	With PNI (*n =* 9)	Without PNI (*n* = 32)	*P* value
Age, mean ± SD, years	63 ± 14	66 ± 9	0.400	66 ± 10	63 ± 9	0.421
Gender (%)			0.891			0.092
Male	14 (60.9%)	45 (59.2%)		8 (88.9%)	22 (68.8%)	
Female	9 (39.1%)	31 (40.8%)		1 (11.1%)	10 (31.2%)	
CEA, median (P25, P75), ng/mL	3.00 (1.16,4.80)	3.29 (1.87,7.12)	0.249	5.10 (3.46,18.87)	2.89 (1.76,8.61)	0.127
CA199, median (P25, P75), ng/mL	9.65 (5.30,15.83)	12.42 (7.55,18.76)	0.335	10.77 (6.71,20.65)	14.26 (8.42,21.51)	0.496
Histologic grade			0.078			0.025
Well differentiated	1 (4.3%)	15 (19.7%)		0 (0)	5 (15.6%)	
Moderately differentiated	18 (78.3%)	53 (69.7%)		6 (66.7%)	25 (78.1%)	
Poorly differentiated	4 (17.4%)	8 (10.5%)		3 (33.3%)	2 (6.3%)	
Primary site			0.763			0.464
Low	8 (34.8%)	28 (36.8%)		1 (11.1%)	8 (25.0%)	
Mid	9 (39.1%)	31 (40.8%)		3 (33.3%)	10 (31.3%)	
High	6 (26.1%)	17 (22.4%)		5 (55.6%)	14 (43.7%)	
Longest diameter, mean ± SD, cm	5.71 ± 2.20	4.71 ± 1.97	0.041	5.22 ± 1.27	4.64 ± 1.90	0.161
mT-stage			< 0.00			0.089
T1/T2	6 (26.1%)	55 (72.4%)	1	4 (44.4%)	24 (75.0%)	
T3	17 (65.4%)	21 (27.6%)		5 (55.6%)	8 (25.0%)	
mLN-status			0.187			0.507
N0	10 (43.5%)	45 (59.2%)		7 (77.8%)	21 (65.6%)	
N1-N2	13 (56.5%)	31 (40.8%)		2 (22.2%)	11 (34.4%)	
CRM			0.149			0.023
Positive	9 (39.1%)	18 (23.7%)		6 (66.7%)	8 (25.0%)	
Negative	14 (60.9%)	58 (76.3%)		3 (33.3%)	24 (75.0%)	
EMVI			0.008			0.637
Positive	11 (47.8%)	15 (19.7%)		3 (33.3%)	8 (25.0%)	
Negative	12 (52.2%)	61 (80.3%)		6 (66.7%)	24 (75.0%)	

*P*-value for each radiomic feature associated with PNI was calculated using Wilcoxon test

*Abbreviations*: *PNI* perineural invasion, *CEA* carcinoembryonic antigen, *CA19–9* carbohydrate antigen 19–9, *mT-stage* MRI reported T-stage, *mLN-status* MRI lymph node-status, *CRM* circumferential resection margin, *EMVI* extramural vascular invasion

### Inter-observer and intra-observer reproducibility of radiomic feature isolation

Based on two measurements of Reader 1, the calculated intra-observer ICC ranged from 0.852 to 0.966. The inter-observer agreement between two readers was within the range of 0.779–0.932. The data suggested favorable intra- and inter-observer reproducibility of feature isolation. Therefore, all outcomes were based on the calculations of Reader 1.

### Feature selection and construction of radiomic model

**Fig. 3:**
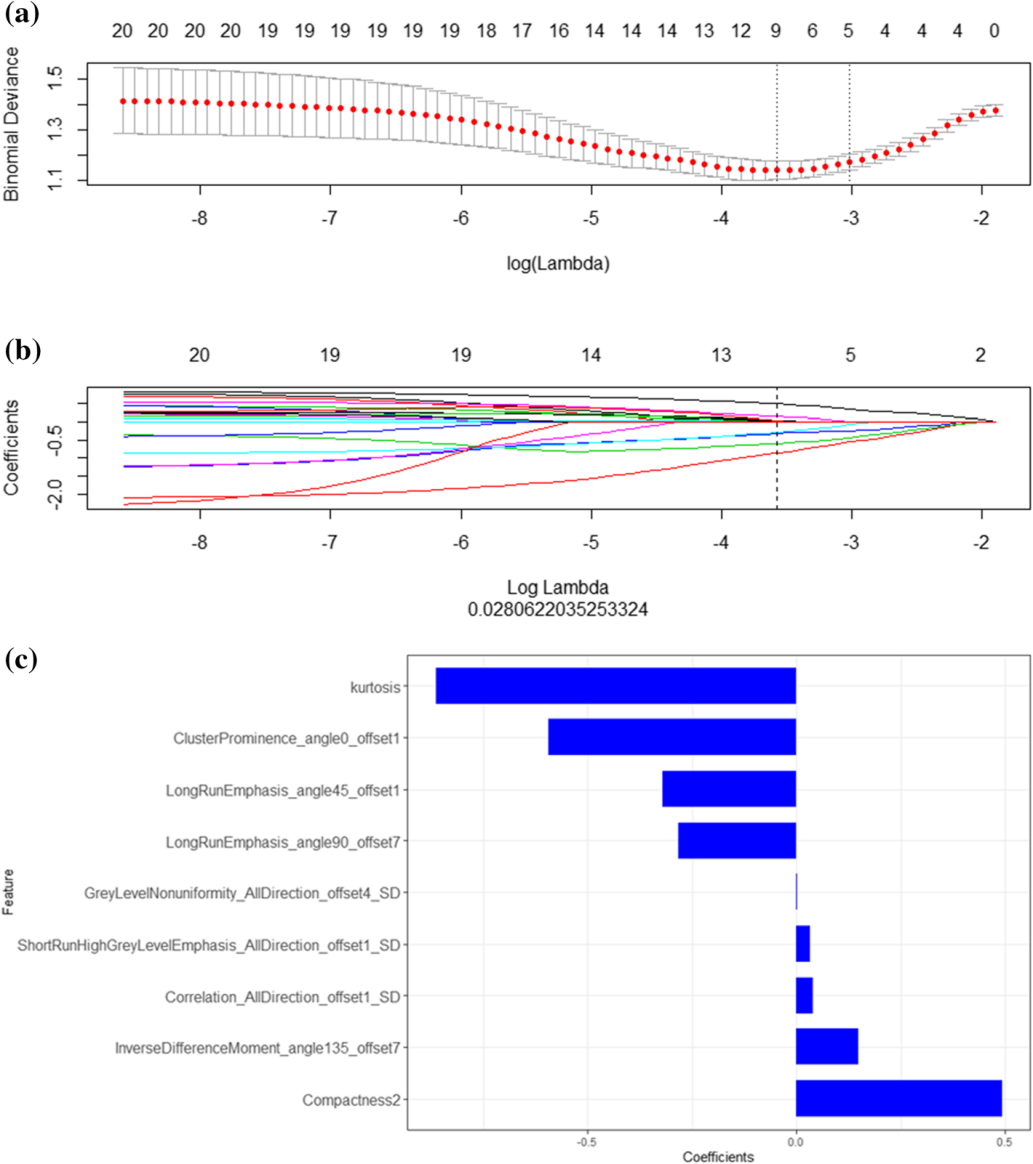
Feature selection and dimensionality reduction. **a** 10-fold cross-validation of the LASSO analysis was performed to select the most valuable features in predicting PNI. The abscissa corresponding to the lowest point of model deviation is the optimal lambda value, that is, the position of the first dashed line. **b** The regression coefficients of LASSO. Each colored line represents the variation curve of the characteristic coefficient with the lambda value. The lambda value (the position represented by the dashed line) found in Fig. 3a is used to determine which parameter has a coefficient that is not 0, then this parameter is used in the final Model building. **c** The final features and corresponding coefficients. The blue bars show corresponding coefficients of each final feature, indicating the importance of PNI prediction

20 features were retained after mRMR selection, then 9 features with non-zero LASSO coefficients were retained (Fig. 3). Rad-score was calculated by summing the weighed coefficients of the chosen features. The final formula of Rad-score in PNI prediction was as follows:

Rad-score = 0.281*GreyLevelNonuniformity_AllDirection_offset1_SD + -0.278*LongRunHighGreyLevelEmphasis_angle90_offset7 + 0.197*LongRunEmphasis_AllDirection_offset1_SD + 0.236*ClusterShade_AllDirection_offset1_SD + 0.333*Compactness2 + 0.119*ShortRunLowGreyLevelEmphasis_AllDirection_offset1_SD + -0.423*Percentile95 + 0.198*GLCMEntropy_AllDirection_offset7_SD + -0.829*ClusterProminence_angle45_offset1 + 0.501*ShortRunEmphasis_AllDirection_offset4_SD + -0.536*LongRunEmphasis_angle45_offset1 + − 0.502*Correlation_AllDirection_offset7_SD + -0.432*LongRunEmphasis_angle90_offset7 + − 0.533.

**Fig. 4:**
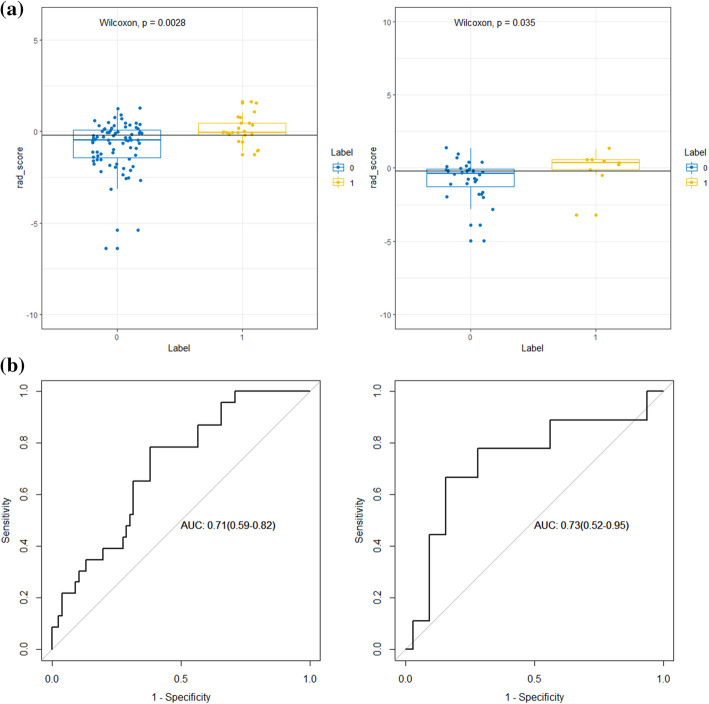
**a** Wilcoxon test of Rad-score between patients with and without PNI. **b** ROC curves of radiomic signature to detect the presence of PNI

Then Wilcoxon test was carried out to compare the Rad-scores between patients with PNI and those without PNI in the training and validation cohorts, respectively (Fig. 4a). The cutoff value was set at 0.408 based on the results. ROC analysis was employed to evaluate the performance of the radiomic model in predicting PNI in the training cohort (AUC, 0.71; 95% confidence interval [CI], 0.59–0.82) and the validation cohort (AUC, 0.73; 95%CI, 0.52–0.95) (Fig. 4b).

### Construction and validation of the predictive nomogram

**Fig. 5:**
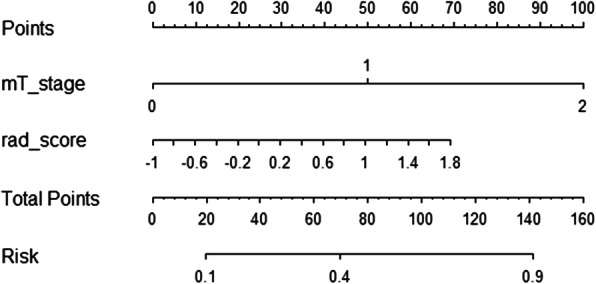
The final nomogram, including Rad-score and mT-stage, in predicting PNI status

**Fig. 6:**
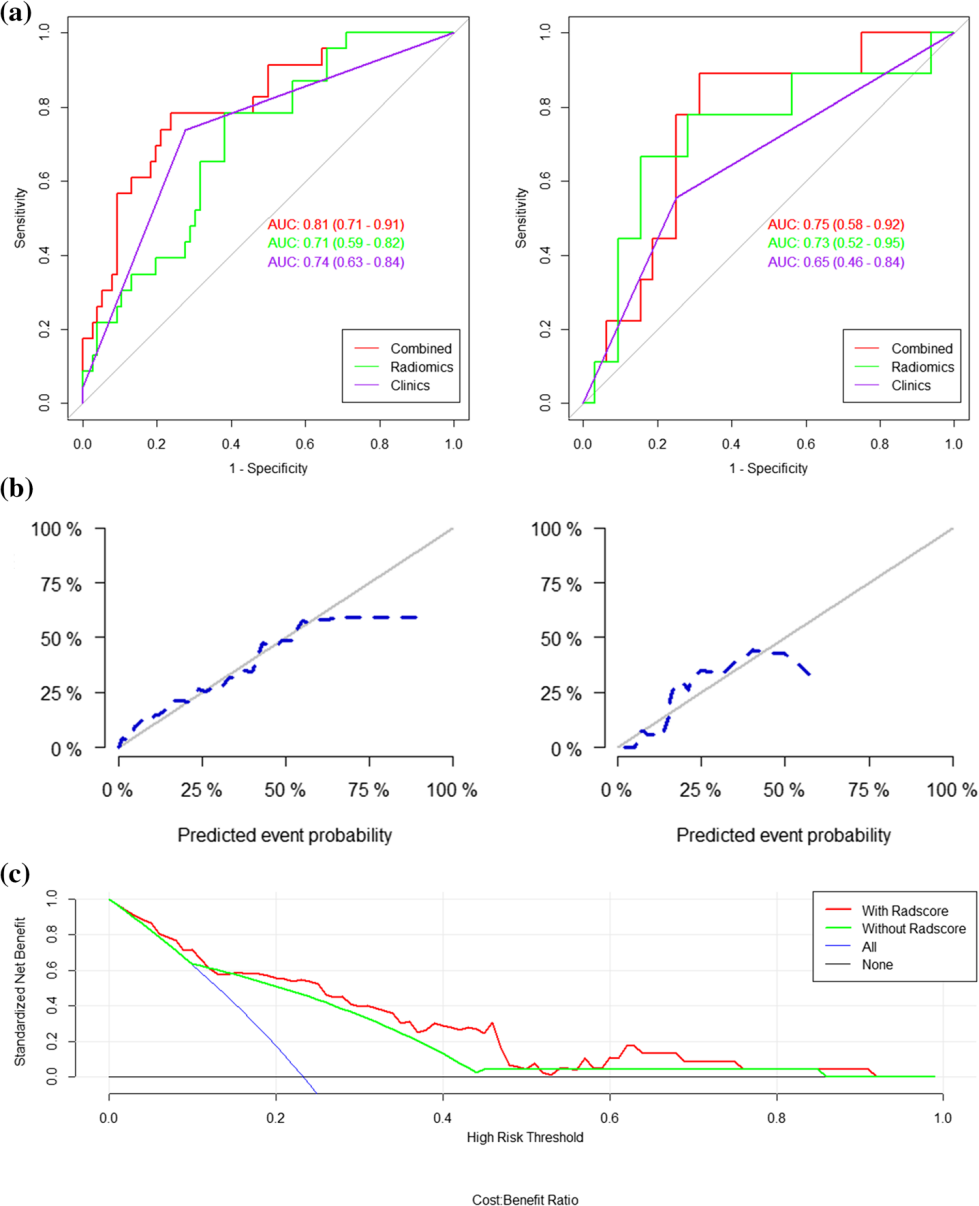
**a** ROC curves of HRMRI findings and radiomic signature to detect the presence of RC PNI in training (left) and validation cohorts (right). **b** Calibration of the radiomic nomogram in training (left) and validation cohorts (right). The solid line represents the reference line with the observed value. The dotted line represents the performance of the hybrid nomogram, while the solid line is corrected for any bias in the hybrid nomogram. **c** DCA was performed to assess the clinical usefulness of the combined model in predicting PNI. The net benefit is measured by the y-axis. The red curves represent the combined model with Rad-score. The green lines represent the model without Rad-score. The blue curves represent all patients with PNI. The black lines represent patients without PNI. It indicated that using nomogram to predict PNI gains more benefit when the threshold probability from 0.05–0.5 and 0.6–0.75 for a doctor or a patient than “treat all” or “treat none”

The nomogram (Fig. 5) was constructed after univariate and multivariate logistic analysis (Tables 1 and 2). Multivariate logistic regression showed us that both mT stage and Rad_score were independent predict factors and mT-stage (OR = 6.39, 95%CI: 2.27–20.36) had a better performance than Rad_score (OR = 2.22, 95%CI:1.22–4.64; P<0.05). The performance of the nomogram was evaluated by ROC curve (AUC = 0.81; 95% CI: 0.71–0.91) and showed a better performance compared with the HRMRI findings only (AUC = 0.74; 95% CI: 0.63–0.84) or Rad-score only (AUC = 0.71; 95% CI, 0.59–0.82) models in training cohort (Fig. 6a). The combined model also exhibited similar performance in the validation cohort. The AUC of the combined nomogram in the validation cohort was 0.75 (95%CI, 0.58–0.92), which was better than HRMRI findings (AUC = 0.65; 95% CI: 0.46–0.84) or Rad-score (AUC = 0.73; 95% CI: 0.52–0.95) only models. Based on the Youden index, the accuracy, sensitivity, and specificity of predicting PNI of RC are shown in Table 3. Hosmer-Lemeshow test was employed to evaluate the consistency between the nomogram and the observed value (*p* = 0.14 for the training cohort, *p* = 0.24 for the validation cohort) (Fig. 6b).

**Table 2:** Univariate and multivariate logistic regression analyses of PNI prediction

Variables	Univariate Logistic		Multivariate Logistic	
	OR (95%CI)	*P* value	OR (95%CI)	*P* value
Histologic grade	2.28 (0.92–6.05)	0.083	–	–
mT-stage	7.41 (2.78–22.62)	0.00015	6.39 (2.27–20.36)	8.02E-04
Longest diameter	1.26 (1.01–1.58)	0.045	–	–
EVMI	3.73 (1.38–10.22)	0.009	–	–
Rad_score	4.23(1.94–10.68)	0.001	2.22 (1.22–4.64)	0.019

*Abbreviations*: *PNI* perineural invasion, *mT-stage* MRI reported T-stage, *EMVI* extramural vascular invasion

**Table 3:** Performance of the model in predicting PNI

	Accuracy	Sensitivity	Specificity	PPV	NPV
Training	0.77	0.50	0.92	0.78	0.76
Validation	0.73	0.44	0.95	0.89	0.69

*Abbreviations*: *PNI* perineural invasion, *PPV* positive predictive value, *NPV* negative predictive value

### Clinical usefulness of the model

Decision curve analysis (DCA) was performed to evaluate the clinical efficiency of the combined model (Fig. 6c). The result indicated that it was clinically useful to use this model in predicting PNI.

## Discussion

PNI is the process by which cancer cells wrap around nerves, and has many associated features, such as a tumor in close proximity to the nerve and involving at least 33% of its circumference, or tumor cells within any of the three layers (endoneurium, perineurium, and epineurium) of the nerve sheath. PNI is an underestimated route of metastatic spread [25, 26]. The existence of PNI greatly affects the local recurrence rate in CRC subjects, resulting in a dramatically decreased survival rate, irrespective of the presence of other adverse factors [5–11]. Moreover, the status of PNI should also be considered a high-risk factor in selecting adjuvant treatment plans [8, 10].

Since PNI has proved to be important in prognosis and treatment plans, the status of PNI is a mandatory content in pathology reports in RC patients in NCCN, ESMO, and Chinese Society of Clinical Oncology (CSCO) guidelines for RC [12–14]. Preoperative and postoperative adjuvant chemotherapy may be a good alternative for PNI-positive subjects [7, 13, 14, 27]. However, it is difficult to recognize PNI through operative imaging, even using HRMRI (Fig. 2) [18, 19].

In our study, 32 patients were confirmed to have PNI, while 108 patients were confirmed to have no PNI. The PNI-positive rate was 22.9% in RC, which was slightly higher than that reported in Knijn N’s meta-analysis (18.2%). This might be related to the retrospective reassessment of the PNI status in our study. Furthermore, we present a nomogram which incorporates mT-stage and radiomic signature, with AUC = 0.81 in the training set and AUC = 0.75 in the validation set. Based on the Youden index, the accuracy, sensitivity, and specificity of the model in predicting PNI of RC were 0.768, 0.500, and 0.921 in the training set and 0.732, 0.444, and 0.957 in the validation set, respectively. Although the sensitivity of this model was relatively low, the specificity and accuracy were high. This could be attributed to the following reasons: postoperative pathology confirmed that only 32 patients had PNI, while 108 patients did not. Hence, we used SMOTE to amplify the small sample data sets and reach a balanced data distribution. However, SMOTE was only used to build the model, and the actual effectiveness of the model was evaluated based on the original samples. This sample bias was large enough that the final model shifted towards the group without PNI. This characteristic also resulted in a high rate of missed diagnosis when the model was used to predict PNI, but a low over-diagnosis rate. In our study, clinical risk factors failed to show an incremental predictive value of PNI.

Alotaibi’s study confirmed that PNI-positive colorectal cancer was associated with higher CEA level, larger tumor size, more advanced pT, pN, and pM categories, poorer differentiation, and a higher rate of positive CRM compared with PNI-negative tumors (*p* = 0.001) [28]. However, there are no logistic analyses to further identify independent predictors. In our study, no statistical difference was observed in the mean preoperative serum CEA level, which is different from Alotaibi’s study. This may be due to following reasons: (a) CEA data in our study were non-normally distributed data, and Wilcoxon test, instead of unpaired Student’s t-test, was used to compare the two groups; (b) our data were obtained within 1 week before operation, which may be different from Alotaibi’s study, for which a data collection time-frame is not indicated; and (c) our study enrolled RC patients rather than CRC patients. Similar to Alotaibi’s study, longest diameter on MR was larger (*p* = 0.041), and the mT stage was advanced (*p* < 0.001) in the PNI-positive group compared with the PNI-negative group. Following the univariate and multivariate logistic regression analyses, the final model included the mT stage and Rad-score. Many recent studies have shown that radiomics and Rad-score can build a reliable bridge between tumor heterogeneity and high-dimensional, mineable quantitative imaging features [15, 16]. In our study, PNI was the target tumor heterogeneity and has great clinical and prognostic significance. We successfully used this newly emerging approach to build a bridge between preoperative HRMRI and the target tumor heterogeneity.

This study has some limitations. (a) Our study is a retrospective analysis, and as only patients who received radical surgery were enrolled, a selection bias might exist. (b) In this study, the PNI status was assessed by H&E staining, rather than by anti-S100 antibody-based immunohistochemistry technique, which facilitated pathologists to diagnose more cases of PNI-positive CRC [5, 29]. Our future study will focus on this favorable technique. (c) The sample bias was large and resulted in low sensitivity. We aim to increase the sample size in the future and believe that increased sample size will substantially improve the model’s sensitivity. (d) All study subjects were enrolled at one hospital and verified internally. Therefore, it is necessary to validate the nomogram using images from other hospitals to increase credibility and robustness.

## Conclusions

Taken together, our study provided a radiomic nomogram that incorporated the radiomic signature and HRMRI findings, which can potentially identify PNI in pre-operative imaging in RC patients.

## Data Availability

The datasets used and/or analysed during the current study are available from the corresponding author on reasonable request.

## Abbreviations

HRMRI: High-resolution magnetic resonance imaging
PNI: Preoperative perineural invasion
RC: Rectal cancer
mT-stage: MRI-reported tumor stage
AUC: Area under the curve
CRC: Colorectal cancer
CRM: Circumferential resection margin
EMVI: Existence of extramural vascular invasion
CEA: Carcinoembryonic antigen
FSE: Fast spin-echo
mLN-status: MRI-reported lymph node status
GLSZM: Gray-level size zone matrix
GLCM: Gray-level co-occurrence matrix
RLM: Run-length matrix
mRMR: Maximum correlation and minimum redundancy
LASSO: Least absolute shrinkage and selection operator
ROC: Receiver operating characteristic
ICC: Intra-class correlation coefficient
SMOTE: Synthetic Minority Oversampling Technique
DCA: Decision curve analysis
LR: Local recurrence
5yCSS: Decreased 5-year cancer-specific survival
5yDFS: 5-year disease-free survival
5yOS: 5-year overall survival
CSCO: Chinese Society of Clinical Oncology
